# Effects of HIV-related worries on fertility motivation moderated by living children among couples living with HIV: A dyadic analysis

**DOI:** 10.3389/fpsyg.2022.1000100

**Published:** 2022-11-10

**Authors:** Yingwu Guo, Yingrong Du, Jun Liu, Jingsong Bai, Jianpeng Gao, Lei Wu, Yongrui Yang, Weibo Wang, Jie Chen, Zhaoyuan Xu, Junfang Yan, Nihong Lu, Chongxi Li, Virasakdi Chongsuvivatwong

**Affiliations:** ^1^Department of Infectious Diseases, Third People’s Hospital of Kunming City, Kunming, China; ^2^Department of Epidemiology, Faculty of Medicine, Prince of Songkla University, Hat Yai, Songkla, Thailand

**Keywords:** dyadic analysis, HIV-related worries, fertility motivation, living children, couples living with HIV

## Abstract

**Introduction:**

HIV-related worries are a major barrier to achieving fertility goals for couples living with HIV (CLWH). We examined the moderating role of living children in the association between HIV-related worries and fertility motivation in CLWH including happiness, well-being, identity, and continuity.

**Methods:**

The data of 322 reproductive-aged CLWH were collected for this cross-sectional study from a referral antiretroviral therapy clinic in Kunming, China between October and December 2020. Intra- and interpersonal mechanisms of association between HIV-related worries and fertility motivation moderated by the number of living children in husband-wife dyads were analyzed by the actor-partner interdependence moderation model.

**Results:**

The high-level HIV-related worries of the wives and husbands were associated with the spouses’ fertility motivation. Having at least one child helped to ameliorate the negative association between one’s own HIV-related worries and fertility motivation. However, there was no evidence of such moderation in the spouse.

**Conclusion:**

Whether the CLWH has at least one living child should be taken into account in counseling. Childless families should be counseled on HIV-related worries as those worries have a greater negative effect on fertility motivation than couples who have a child.

## Introduction

It was anticipated that by the end of 2021, fertility issues would impact 3.8% of reproductive-aged adults living with HIV worldwide (Global HIV and AIDS statistics—Fact sheet, 2022). This represents a growing public need to address this issue. A systematic review reported a 42.04% pooled prevalence of fertility desire among people living with HIV (Yan et al., 2021). Furthermore, fertility motivation is a critical contributor to the occurrence of fertility desire. Fertility motivation has been defined as the disposition to react positively or negatively to childbearing, which has changed dramatically throughout time (Miller, 1994; Dyer et al., 2008). The findings of Miller et al. demonstrated that an increased positive motive for childbearing enhances fertility desire (want to have a child) (Miller and Pasta, 1995). HIV-related worries are one of the most prevalent forms of psychological suffering among couples living with HIV (CLWH) (CBD et al., 2019). Consistently, health services to safely help CLWH to have a new child is available in China, such as the National Free ART program and prevention of mother-to-child HIV transmission (PMTCT). Yet HIV-related worries of CLWH on fertility are still an unsolved problem.

A previous study found that rural Malawians, especially HIV-positive men, want fewer children. Women fear the health repercussions of HIV-positive pregnancies and childbearing, while men consider childbirth fruitless since they predict their own early death and the deaths of their future offspring (Yeatman, 2009). Achieving a broad consensus regarding HIV and fertility is one of the most essential tasks HIV-positive couples must acquire. Both HIV-related and reproductive factors are strongly associated with fertility outcomes such as motivation and behavior (Nattabi et al., 2009; Joseph Davey et al., 2018; Siegel et al., 2018; Yan et al., 2021). Recent research suggested that fertility issues surrounding worry about the risk of HIV transmission and fertility are correlated (Haddad et al., 2017; Marston et al., 2017; Milford et al., 2021). HIV-related restrictions on a couple’s fertility are alarming given that many HIV-positive couples report experiencing difficulties (Rogers et al., 2016).

Fertility motivation is a crucial moment in the process of establishing a family plan for a couple living with HIV. HIV-related worries experienced early in reproductive decision-making were associated with later life span extension due to the efficacy of antiretroviral therapy (ART) (Faraji et al., 2021), which suggests that HIV-related fertility hurdles may have long-term repercussions in CLWH. Other kinds of worries, including antiretroviral drug toxicity (Joseph Davey et al., 2018) and stigma or discrimination (Turan and Nyblade, 2013), are positively linked to fertility behavior.

From a previous study in China, 66.9% of households in the HIV context had two to three children, while 21.4% had one child (Ji et al., 2007). Having living children is a source of realizing reproductive goals. Various investigations have shown that reproductive desires are complicated and contradictory which reflect conflicts between the family and social expectations to have children and pressures to avoid HIV infection and reinfection (Finocchario-Kessler et al., 2010; Wekesa and Coast, 2014; Kimani et al., 2015). In the general population, having children is known to be a determinant of fertility motivation in the family (Irani and Khadivzadeh, 2018). However, although the number of living children is a unique measure of a couple’s fertility (Moshoeshoe and Madiba, 2021), whether having a child can reduce the effect of HIV-related worries on fertility motivation has not been well examined. Fertility planning in CLWH needs to occur in the context of a dyad. Fertility motivation in CLWH is therefore a dyad variable to be measured on both sides of CLWH. On the other hand, HIV-related worries which are known to be one’s own fertility desire may also influence that of the spouse (Cook et al., 2014).

This dyad complex is more complicated by the fact that a stable couple shares the same set of children, whose presence may moderate the effect of HIV-related worries on fertility motivation. A statistical analysis procedure that accounts for non-independence is essential. Kenny et al. devised the actor-partner interdependence moderation model (APIMoM) to solve this problem (Acitelli et al., 2013; Davey et al., 2018; Stas et al., 2018). APIMoM can simultaneously examine the relationship of the variable on husband-and-wife sides as well as investigate the influence of the present living children, which is a shared variable for CLWH. In this study, we adjusted this APIMoM model to understand how the complex psychology of the CLWH interacts with the couple. In this research, we aimed to examine the effects of HIV-related worries on fertility motivation in CLWH. At the same time, we wanted to document the moderating effect of when there is at least one living child in the dyad relationship. Such understanding will help health care providers of CLWH to fine-tune the counseling plan to suit the needs of couples with and without a child.

## Materials and methods

### Study design

This was a cross-sectional study on CLWH attending the ART clinic of the referred hospital in the Kunming City of China.

### Participants and data collection procedure

The principal investigator contacted the managers of ART clinics to request their permission for their patients to participate in this study. Our research comprised reproductive-aged PLWH in a stable, sexually active heterosexual relationship with no more than one kid who had received ART for more than a year. (1) Not speaking Chinese well; (2) Having a chronic ailment; (3) Infertility (e.g., history of hysterectomy, oophorectomy, vasectomy). The consent of an HIV-positive participant was the primary respondent. He/she was asked to let the researcher contact his/her spouse for an interview. The couple who consented was then using the same questionnaire separately interviewed in a face-to-face meeting in a private place at the hospital or *via* telephone. The one exception was that the spouse would not be asked whether or not they had living children to avoid interfering with each other. The interview lasted between 15 and 30 min.

### Sample size estimation

A sample size in this range might guarantee that the variance between the calculated sample and the population parameter is steady and modest. Based on the Structural Equation Model Sample Size Calculator (Christopher, 2010; A-priori Sample Size for Structural Equation Models References – Free Statistics Calculators, 2021), we used this calculator to determine the sample size needed for the research using a structural equation model (SEM) with 39 observable variables, 12 latent variables, an expected effect size of 0.3, type I error at 0.05 and the desired statistical power levels of 0.8. The calculation indicated that the minimum sample size is necessary to detect specified effect was 200.

### Application of actor-partner interdependence moderation model in this study

**Figure 1:**
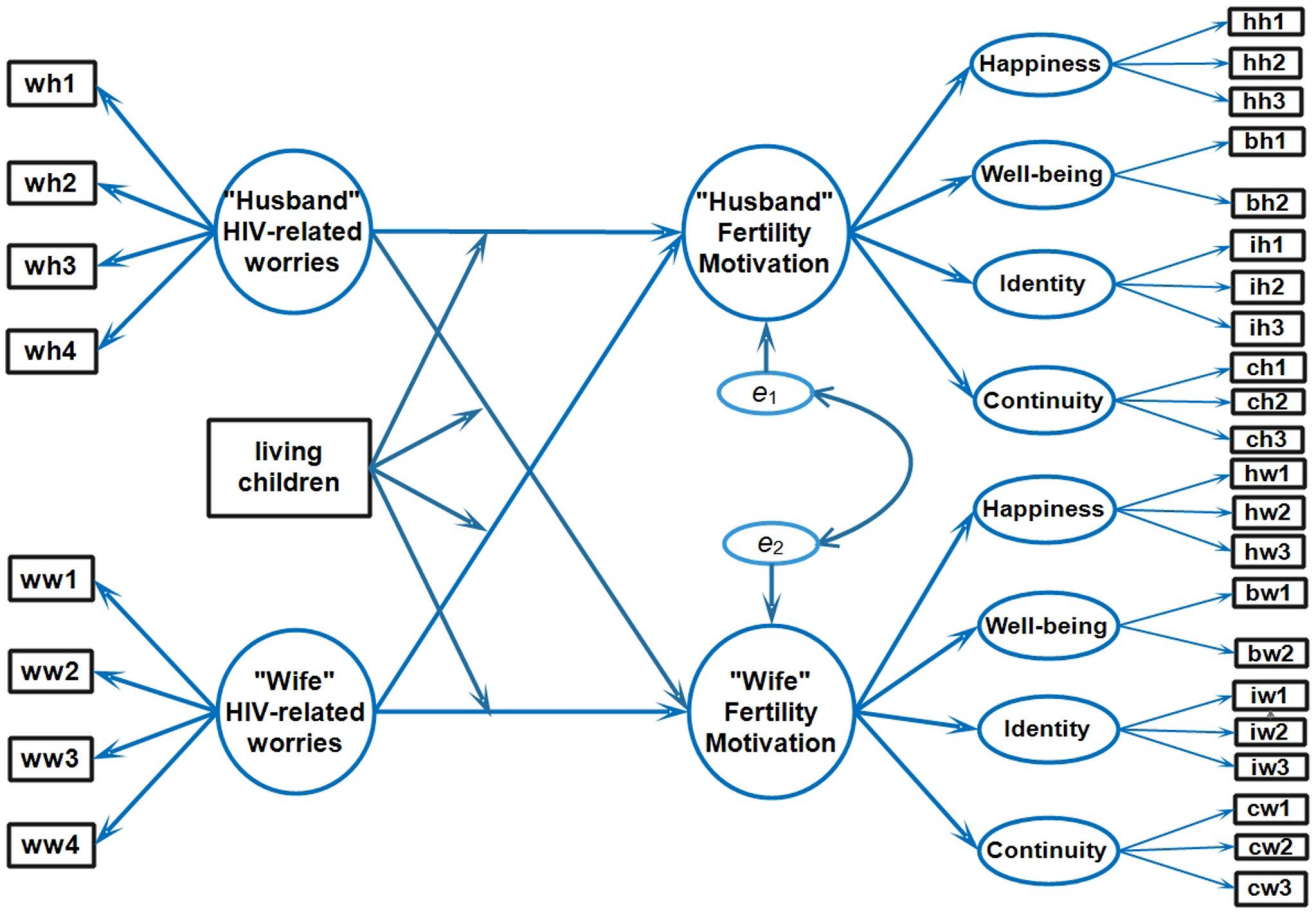
Conceptual actor-partner interdependence moderation model. wh1/ww1, wh2/ww2, wh3/ww3, wh4/ww4, Worries indicators; hh1/hw1, hh2/hw2, hh3/hw3, Happiness indicators; bh1/bw1, bh2.bw2, Well-being indicators; ih1/iw1, ih2/iw2, ih3/iw3, Identity indicators; ch1/cw1, ch2/cw2, ch3/cw3, Continuity indicators. The individual definitions of each abbreviation were explained in Supplementary Tables 1, 2.

The application of APIMoM is illustrated in Figure 1. The husband and the wife comprise a dyadic unit. Each had his/had own pathway of a causal relationship between HIV-related worries and fertility motivation. Additionally, each acted as an “actor” whose worries cross-influenced his spouse or “partner.” These direct and cross-influences were “moderated” (modified) by the number of living children.

### Measures

#### HIV-related worries

The HIV-related worries list was measured by Djiometio et al. (2019). A 4-item scale was used to assess HIV-related worries (Supplementary Table 1). On a 5-point Likert-type scale the respondents rated the items that ranged from 1 (strongly disagree) to 5 (strongly agree). After recording the negative items, the possible total scale scores ranged from 4 to 20 with higher scores reflecting greater levels of anxiety. Cronbach’s alpha coefficients for the scales of husband and wife in this study were 0.77 and 0.86, respectively.

#### Fertility motivation

The scale for fertility motivation was adjusted from van Balen and Trimbos-Kemper (1995). The fertility subscale of the CLWH scale was used to evaluate fertility motivation (Supplementary Table 2). Fertility motivation consisted of 11 statements representing four components recognized and labeled as happiness (three items), well-being (two items), identity (three items), and continuity (three items). On a 5-point Likert scale, responses ranged from 1 (strongly disagree) to 5 (strongly agree or definitely agree). The possible scores on both subscales ranged from 11 to 55. Higher scores suggested stronger fertility motivation. The Chinese version of the fertility motivation questionnaire was validated and is frequently used among HIV-positive individuals in the pilot study. A principal component analysis using a varimax rotation in the basic structure generated four components with eigenvalues >0.5 that accounted for 85% of the variance. Cronbach’s alpha values for the fertility of the husbands and wives in the reliability tests of this study were 0.81 and 0.96, respectively.

#### Number of living children

We asked the respondents whether the couple had any living children. The dummy variable coded as 0 if the couple had no children and 1 otherwise (regardless of which parent was the primary respondent). For the ethical reason, we did not attempt to solve any discrepancy in the answer to avoid any conflicts within the couple.

### Statistical analysis procedure

This APIMoM was conducted using the lavaan package in R (Rosseel, 2012). The main pathways were the relationship between HIV-related worries and fertility motivation with self and crossing to the partner (spouse). The moderation terms included whether the CLWH had living children and whether HIV status was discordant. Structural equation modeling (SEM) was used to estimate these effects (Kenny and Ledermann, 2010).

To ensure that the SEM was valid, the Pearson correlation matrix of the HIV-related worries and fertility motivation scale was computed. Cronbach alpha values of each subdomain (HIV-related worries, well-being, happiness, identity, etc.). We first conducted confirmatory factor analyses (CFA) (Brown, 2006). Then we employed SEM using maximum likelihood estimation (Lam and Maguire, 2012). Multiple fit indices, as proposed by the literature, were used to assess the overall model fit. Five goodness-of-fit indices were used: (A) chi-square/degree of freedom test (χ2 /df); (B) comparative fit index test (CFI); (C) Tucker-Lewis Index test (TLI); (D) standardized root mean square residual test (SRMR); and (E) root mean square error of approximation (RMSEA) (Maydeu-Olivares, 2017; Shi and Maydeu-Olivares, 2020; Comparative Fit Indexes in Structural Models – PsycNET, 2022).

## Results

### Characteristics of the couples living with HIV

Of 322 study couples, 28.9% (93) were both HIV positive, 31.1% (100) had only an HIV test on the husband’s side and 68% (129) only on the wife. The average (SD) ages of husbands and wives were 37.3 (6.37) and 33.95 (5.22) years, respectively. In the husband group, the most frequent educational level was senior high school or less, whereas, in the wife group, the most common educational level was junior high school or less. The percentage of men with a graduate degree or higher was greater than the proportion of wives among all husbands and wives with a graduate degree or higher (17.7%). When questioned about their job status, many wives in the study said they were unemployed. Many respondents were of Han ethnicity and dwelled in rural areas. Many couples (68%) indicated New Rural Cooperative Medical Insurance as their supplier of medical insurance and Urban Residents Basic Medical Insurance. Table 1 provides more information about the characteristics of the sample population.

**Table 1:** Socio-demographic of couples living with HIV.

	Husband (*N* = 322) *n* (%)	Wife (*N* = 322) *n* (%)	Total (*N* = 644) *n* (%)
**Age categories**			
20–30	32 (9.9)	73 (22.7)	105 (16.3)
31–35	94 (29.2)	102 (31.7)	196 (30.4)
36–40	93 (28.9)	105 (32.6)	198 (30.7)
41+	103 (32)	42 (13)	145 (22.5)
**Education level**			
Primary school	34 (10.6)	45 (14)	79 (12.3)
Junior school	95 (29.5)	125 (38.8)	220 (34.2)
Senior school	107 (33.2)	95 (29.5)	202 (31.4)
Graduate and above	86 (26.7)	57 (17.7)	143 (22.2)
**Registered residence**			
Rural	199 (61.8)	209 (64.9)	408 (63.4)
Urban	123 (38.2)	113 (35.1)	236 (36.6)
Ethnic group			
Han	290 (90.1)	266 (82.6)	556 (86.3)
Others	32 (9.9)	56 (17.4)	88 (13.7)
**Occupation status**			
Jobless	49 (15.2)	107 (33.2)	156 (24.2)
Manual laborer	46 (14.3)	25 (7.8)	71 (11)
Private employee	75 (23.3)	89 (27.6)	164 (25.5)
Self-employed	108 (33.5)	83 (25.8)	191 (29.7)
Government employee	44 (13.7)	18 (5.6)	62 (9.6)
**Medical insurance status** ^a^			
NRCMS	186 (57.8)	196 (60.9)	382 (59.3)
UEBMI	35 (10.9)	22 (6.8)	57 (8.9)
URBMI	101 (31.4)	104 (32.3)	205 (31.8)

URBMI, Urban Residents Basic Medical Insurance; UEBMI, Urban Employees Basic Medical Insurance; NRCMS, New Rural Cooperative Medical Insurance Scheme.

a Chinese law established universal medical insurance schemes.

### Pearson correlation and confirmatory factor analysis

The correlation of fertility motivation between the husbands and wives was high (*r* = 0.65, *p* < 0.01). This correlation suggested a sufficient overlap of the scores between the husbands and wives on fertility motivation which allowed us to consider the dyad as the unit of analysis. Table 2 reports all other correlations between the variables. Pearson’s correlation analysis showed that significant and positive correlations were found between HIV-related worries and fertility motivation in both the husbands and their wives (*r* = −0.18–0.45, *p* < 0.01). In this study, all scales had good Cronbach’s *α* reliability (CR = 0.77–0.94) and average variance extracted discriminant validity coefficients (0.49–0.79).

**Table 2:** Descriptive statistics and bivariate correlation in husband-wife dyads.

	Mean (SD)	1	2	3	4	5	6	7	8	9	10	11	12
Happiness (H)	3.9 (0.64)	−											
Well-being (H)	3.4 (0.86)	0.58**	−										
Identity (H)	4.2 (0.51)	0.50**	0.66**	-									
Continuity (H)	4.4 (0.59)	0.32*	0.43*	0.37*	−								
Happiness (W)	3.9 (0.59)	0.49	0.39*	0.33*	0.22	−							
Well-being (W)	3.9 (0.76)	0.40*	0.57**	0.45**	0.29	0.62**	−						
Identity (W)	4.2 (0.51)	0.31*	0.41**	0.63**	0.23	0.49**	0.66**	−					
Continuity (W)	4.5 (0.62)	0.14	0.19	0.16*	0.39	0.22	0.29*	0.23	−				
HIV-related worries (H)	−0.75 (0.80)	−0.2**	−0.26**	−0.22*	−0.14*	−0.14*	−0.19	−0.15*	−0.07	−			
HIV-related worries (W)	−0.57 (0.84)	−0.19*	−0.25**	−0.22*	−0.14	−0.34**	−0.46**	−0.36**	−0.16	0.13	−		
Fertility motivation (H)	4.1 (0.44)	0.66**	0.88**	0.75**	0.49**	0.44**	0.60**	0.47**	0.21	−0.30*	−0.3**	−	
Fertility motivation (W)	4.0 (0.45)	0.43*	0.58**	0.49**	0.32	0.68**	0.91**	0.72**	0.32	−0.21*	−0.5**	0.65**	−
CR	−	0.87	0.88	0.88	0.94	0.81	0.83	0.89	0.96	0.86	0.77	0.87	0.86
AVE	−	0.72	0.77	0.59	0.56	0.75	0.79	0.67	0.49	0.65	0.67	−	−

SD, standard deviation; H, husband; W, wife; CR, Cronbach alphas; AVE, average variance extracted.

*p < 0.05; **p < 0.01.

### Actor-partner interdependence moderation model results

**Figure 2:**
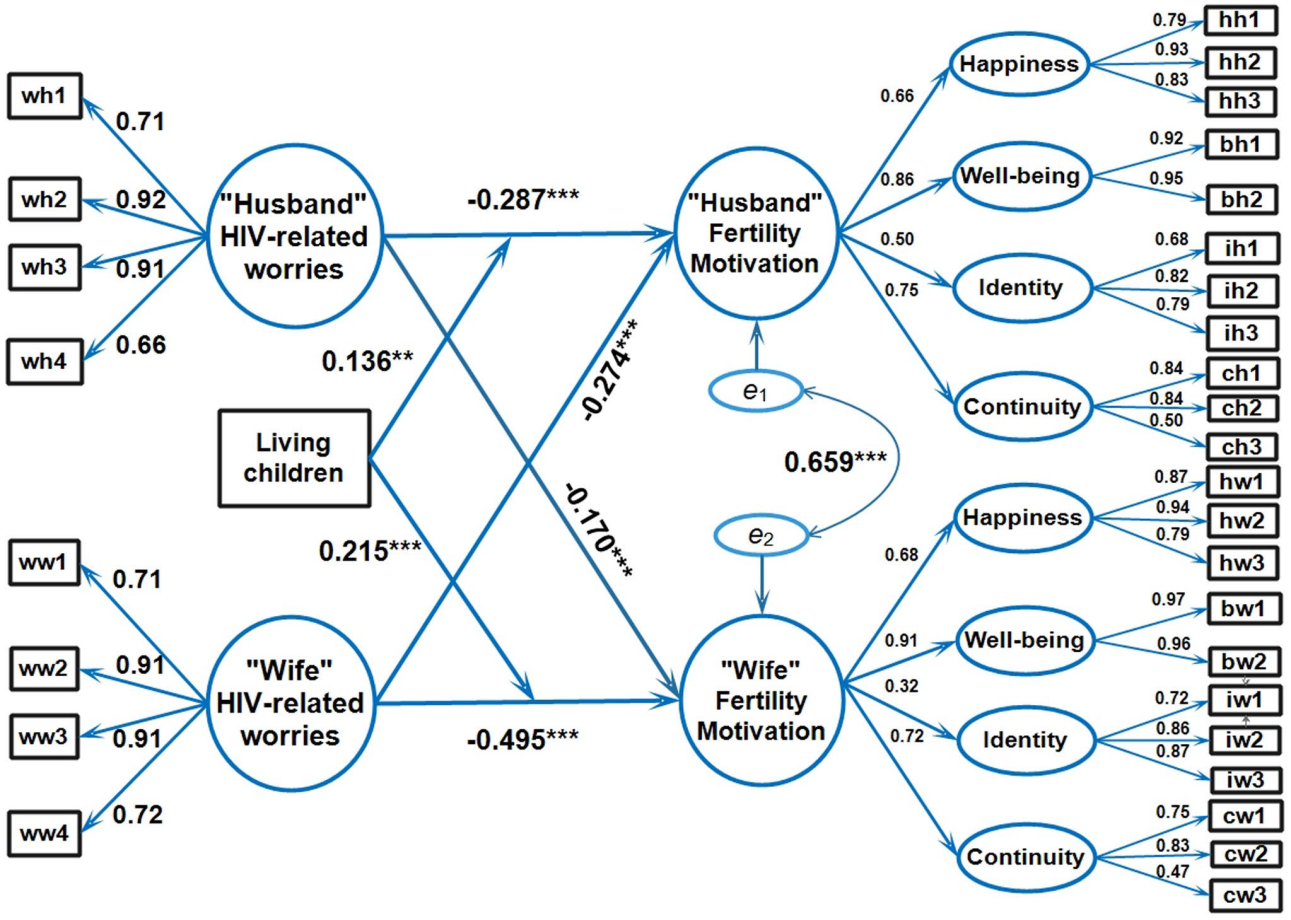
Actor-partner interdependence moderation model of husband-wife dynamics (*N* = 322). wh1/ww1, wh2/ww2, wh3/ww3, wh4/ww4, Worries indicators; hh1/hw1, hh2/hw2, hh3/hw3, Happiness indicators; bh1/bw1, bh2.bw2, Well-being indicators; ih1/iw1, ih2/iw2, ih3/iw3, Identity indicators; ch1/cw1, ch2/cw2, ch3/cw3, Continuity indicators. The individual definitions of each abbreviation were explained in Supplementary Tables 1, 2.

One APIMoM was conducted to assess the main effect of HIV-related worries on fertility motivation factors. The model had a good fit: *χ*^2^/*df* = 2.08; CFI = 0.928; TLI = 0.918; RMSEA = 0.059; and SRMR = 0.067. The explained variance of fertility motivation in husband-wife dyads through HIV-related worries was 37.9% and 51%, respectively (Figure 2). All covariates were included in the model (Table 2). On each side of the couple, the latent variables, and fertility motivation were positively explained by happiness, well-being, identity, and continuity. HIV-related worries also had significant and negative effects on the fertility motivation of the spouse to a smaller degree than on one’s own HIV-related worries. When we added “any living child” as the moderator, all covariates were included in the model (Figure 2). Regarding actor impacts, husbands and wives who had HIV-related worries were adversely motivated by their fertility (*β* = −0.287, *p* < 0.001 and *β* = −0.495, *p* < 0.001, respectively). Concerning relationship impacts, men with more anxious wives reported reduced reproductive motivation (*β* = −0.274, *p* < 0.001). This effect was smaller than HIV-related worries of the husband on the wife’s fertility motivation (*β* = −0.170, *p* < 0.001). Since the core APIMoM was saturated, goodness-of-fit indices were explained Having at least one child positively moderated the negative HIV-related worries on the same individual. This effect was stronger on the husband’s part (*β* = 0.215, *p* < 0.001) than on the wife’s part (*β* = 0.136, *p* < 0.001). The moderator, however, had no significant effect on the relationship between the subject’s HIV-related worries and his/her spouse’s fertility motivation. In other words, the presence of at least one child moderated the negative effect of one’s own HIV-related worries but not the spouse’s fertility motivation.

## Discussion

In this paper, our discussion is centered on the following: (A) There was a high correlation of fertility motivation between husband and wife; (B) HIV-related worries have negative effects on fertility motivation in people living with HIV but also in the spouse, and (C) Having at least one child moderates this negative effect in the individual but not in the spouse.

Our data showed that stable CLWH was a unit of dyad as the intra-couple fertility motivation had a high correlation coefficient. This was consistent with a prior study that found CLWH often had a common motivation for children and that having a motivated spouse was the largest predictor of a participant’s motivation to have more children (Pintye et al., 2015). Spéder and Kapitány (2009) found that happier men and women prefer having children sooner. The impact of happiness on childbearing intentions varies. Aassve et al. (2016) found that women’s satisfaction seemed to have a larger role in the choice to have a second child. People who are optimistic and content with their life path and future prospects are more likely to fulfill their fertility goals. To have a child, a satisfying relationship should be sought. Berninger et al. (2011) also observed that in West Germany, the quality of a positive relationship relates to reproductive intention. Sebert Kuhlmann et al. (2019) indicated, however, that women who experience intimate partner abuse are less likely to want more children. The finding implies that services for fertility planning should be provided to CLWH as a dyad and not on an individual basis.

Cross effects of HIV-related worries on a spouse’s fertility motivation could be explained by inter-dependence within the CLWH. Adverse effects on the spouse would have an important impact on the individual’s well-being. Again, this emphasized the importance of CLWH-based counseling. Both the effect of HIV-related worries on one’s own fertility motivation and cross effects on the spouse were stronger in the wife (*β* = −0.495, *p* < 0.001 and *β* = −0.274, *p* < 0.001, respectively) than in the husband (*β* = −0.287, *p* < 0.001 and *β* = −0.170, *p* < 0.001, respectively). Thus, the wife needs stronger psychological support than the husband to alleviate the effect of HIV-related worries on fertility motivation. Negative effects from the wife on the husband were stronger than from husband to wife. This was possibly due to reproductive physiology, especially pregnancy, and household welfare that are mainly shouldered by the wife (Allendorf, 2010; Rahman et al., 2018; Lee et al., 2021).

Our last finding was that having at least one child can reduce (moderate) the unwanted effect of HIV-related worries on fertility motivation. This is consistent with Kipp et al. (2011) who proposed that having children may be a way to alleviate HIV-related worries during fertility decision-making that results in an increased drive to have more children. There is a possibility that having children is a valid need for changing linkages between HIV-related worries and fertility motivation but having no children may be less successful. Milford et al. reported comparable findings (Milford et al., 2021) that the ability of CLWH to share problem-solving skills assisted them in avoiding HIV-related worries. For this reason, women with just one child are more adaptable in the face of HIV-related worries, since their fertility motivations are more likely to fluctuate (Mynarska and Rytel, 2020). This flexibility may help them adjust to HIV-related worries. Although HIV-related worries had substantial impacts on reproductive motivation in the immediate aftermath, having had at least one child partially alleviated the problem. Interestingly, we have examined the possibility of such moderation on the cross-spouse adverse effect of HIV-related worries and found this was not significant. This can be explained by the fact that the level of cross-spouse effect was already low although statistically significant. The implication of this is that CLWHs without any children should receive more intensive counseling on HIV-related worries than couples with at least one child (Rogers et al., 2016).

### Limitations

The data were obtained from CLWH with the consent of both the husband-and-wife for the interviews. The high level of cross-spouse correlation and the effect of at least one living child may not be generalized directly to CLWH with less marital harmony. Despite this limitation, the findings may be useful for fertility counseling in CLWH who have a good marital relationship and are ready to conceive.

## Conclusion

HIV-related worries of the PLWH negatively affect his/her own and the spouse’s fertility motivation. This effect is moderated by having at least one child. This information should be taken into account on fertility counseling for the CLWH.

## Data availability statement

All pertinent information is contained inside the text and its accompanying information files.

The datasets presented in this article are not readily available because according to the regulations of China CDC on HIV/AIDS patient management, patient information shall not be uploaded and disclosed. Requests to access the datasets should be directed to 568606564@qq.com

## Ethics statement

The studies involving human participants were reviewed and approved by the Ethics Committee of Prince of Songkla University (REC-63-208-18-1) and the Research Ethics Review Committee of the Third People’s Hospital (2020072001). In this study, pseudonyms were used to protect the identity of the participants. Written informed consent for participation was not required for this study in accordance with the national legislation and the institutional requirements.

## Author contributions

YG and VC: conceptualization, methodology, project administration, and validation. YG, YD, JB, and WW: data curation. YG, VC, and JL: formal analysis. YG and JL: funding acquisition. YG, YD, JL, JB, and WW: investigation. YD, JL, LW, JG, WW, JC, ZX, JY, NL, and CL: resources. VC: supervision and writing—review and editing. YG: writing—original draft. All authors contributed to the article and approved the submitted version.

## Funding

Funding was received from the Higher Education Research Promotion and Thailand’s Education Hub for the Southern Region of ASEAN Countries Project Office of the Higher Education Commission (TEH-AC:016/2018).

## Conflict of interest

The authors declare that the research was conducted in the absence of any commercial or financial relationships that could be construed as a potential conflict of interest.

## Publisher’s note

All claims expressed in this article are solely those of the authors and do not necessarily represent those of their affiliated organizations, or those of the publisher, the editors and the reviewers. Any product that may be evaluated in this article, or claim that may be made by its manufacturer, is not guaranteed or endorsed by the publisher.

## Author disclaimer

The opinions expressed in this viewpoint are solely those of its writers and do not necessarily reflect the opinions of the institutions with which they are connected.

## Abbreviations

AIDS: acquired immunodeficiency syndrome
ART: antiretroviral therapy
APIMoM: actor-partner interdependence moderation model
CLWH: couples living with HIV
CFI: comparative fit index
HIV: human immunodeficiency
RMSEA: root mean square error of approximation
SRMR: standardized root mean square residual
SEM: structural equation model
TLI: Tucker-Lewis Index
*χ*^2^/df: chi-square/degree of freedom
