# Supplementary material for: Effects of HIV-related worries on fertility motivation moderated by living children among couples living with HIV: A dyadic analysis
Source: Front Psychol. 2022 Nov 10;13:1000100. doi: 10.3389/fpsyg.2022.1000100 (PMC9685432; doi:10.3389/fpsyg.2022.1000100)
Supplement: Supplementary file 1 [file Data_Sheet_1.docx]

**APPENDIX TABLE 1.** Structural constructs of HIV-related worries

| **Covariates** | **Items** | **Description of variables** |
| --- | --- | --- |
| If I have a child, I would be worried that…. | **wh1/ww1** | I would be unable to care for the children until they are adults. |
|  | **wh2/ww2** | My HIV medication will affect my ability to care about my child. |
|  | **wh3/ww3** | I would not focus enough on my medication. |
|  | **wh4/ww4** | My children would suffer discrimination at school. |

**APPENDIX TABLE 2.** Structural constructs of fertility motivation

| **Subscale Items** | **Indicator** | **Constructs** |
| --- | --- | --- |
| It is nice to have children around | hh1/hw1 | Happiness |
| To have a unique relationship with the child | hh2/hw2 |  |
| Bringing up children brings happiness | hh3/hw3 |  |
| Children make life complete | bh1/hw1 | Well-being |
| It gives you a goal to live for | bh2/hw2 |  |
| It is obvious to have children | ih1/iw1 | Identity |
| It is a sign of being grown-up | ih2/iw2 |  |
| It is the nature of man/woman | ih3/iw3 |  |
| To continue my bloodline into the next generation | ch1/cw1 | Continuity |
| To continue the family name | ch2/cw2 |  |
| My family expect continuing the family line of me | ch3/cw3 |  |
